# How does it feel? An exploration of neurobiological and clinical correlates of alexithymia in trauma-exposed police-officers with and without PTSD

**DOI:** 10.1080/20008066.2023.2281187

**Published:** 2023-11-21

**Authors:** Cindy van Sleeuwen, Mirjam van Zuiden, Saskia B. J. Koch, Jessie L. Frijling, Dick J. Veltman, Miranda Olff, Laura Nawijn

**Affiliations:** a Department of Psychiatry, Amsterdam Neuroscience, Amsterdam Public Health, Amsterdam UMC, Location University of Amsterdam, Amsterdam, the Netherlands; b Donders Institute for Brain, Cognition and Behavior, Centre for Cognitive Neuroimaging, Radboud University, Nijmegen, the Netherlands; c Department of Psychiatry and Medical Psychology, OLVG Hospital, Amsterdam, the Netherlands; d Department of Psychiatry, Amsterdam Neuroscience, Amsterdam Public Health, Amsterdam UMC, Location Vrije Universiteit Amsterdam, Amsterdam, the Netherlands; e Arq National Psychotrauma Centre, Diemen, the Netherlands

**Keywords:** Alexithymia, posttraumatic stress disorder, trauma, oxytocin, amygdala, insula, Alexitimia, trastorno de estrés postraumático, trauma, oxitocina, amígdala, ínsula, 述情障碍, 创伤后应激障碍, 创伤；催产素；杏仁核；脑岛

## Abstract

**Background:** Alexithymia, an inability to recognise one’s emotions, has been associated with trauma-exposure and posttraumatic stress disorder (PTSD). Previous research suggests involvement of the oxytocin system, and socio-emotional neural processes. However, the paucity of neurobiological research on alexithymia, particularly in trauma-exposed populations, warrants further investigation.

**Objective:** Explore associations between alexithymia, endogenous oxytocin levels, and socio-emotional brain function and morphometry in a trauma-exposed sample.

**Method:** Dutch trauma-exposed police officers with (*n* = 38; 18 females) and without PTSD (*n* = 40; 20 females) were included. Alexithymia was assessed with the Toronto Alexithymia Scale (TAS-20). Endogenous salivary oxytocin was assessed during rest, using radioimmunoassay. Amygdala and insula reactivity to socio-emotional stimuli were assessed with functional MRI, amygdala and insula grey matter volume were derived using Freesurfer.

**Results:** Alexithymia was higher in PTSD patients compared to trauma-exposed controls (*F*(1,70) = 54.031, *p* < .001). Within PTSD patients, alexithymia was positively associated with PTSD severity (*ρ*(36) = 0.497, *p* = .002). Alexithymia was not associated with childhood trauma exposure (*β* = 0.076, *p* = .509), police work-related trauma exposure (*β* = −0.107, *p* = .355), oxytocin levels (*β* = −0.164, *p* = .161), insula (*β* = −0.170, *p* = .158) or amygdala (*β* = −0.175, *p* = .135) reactivity, or amygdala volume (*β* = 0.146, *p* = .209). Insula volume was positively associated with alexithymia (*β* = 0.222, *p* = .016), though not significant after multiple testing corrections. Bayesian analyses supported a lack of associations.

**Conclusions:** No convincing neurobiological correlates of alexithymia were observed with any of the markers included in the current study. Yet, the current study confirmed high levels of alexithymia in PTSD patients, independent of trauma-exposure, substantiating alexithymia’s relevance in the clinical phenotype of PTSD.

## HIGHLIGHTS

- Little is known about neurobiological correlates of alexithymia in trauma-exposed and posttraumatic stress disorder (PTSD) populations.
- In this highly trauma-exposed sample, alexithymia was associated with PTSD symptoms, but not with childhood or adult trauma exposure, suggesting alexithymia is not a direct consequence of trauma.
- Alexithymia was not convincingly associated with salivary oxytocin, amygdala and insula reactivity to socio-emotional stimuli, amygdala or insula grey matter volume.

## Abstract (es)

**Antecedentes:** La alexitimia, la incapacidad para reconocer las emociones propias, se ha asociado con exposición a trauma y al trastorno de estrés postraumático (TEPT). Las investigaciones previas sugieren el compromiso del sistema de oxitocina y de procesos neuronales socioemocionales. No obstante, la escasez de estudios en la neurobiología de la alexitimia, particularmente en poblaciones expuestas a trauma, genera demanda de mayor investigación en el área.

**Objetivo:** Explorar las asociaciones entre la alexitimia, los niveles endógenos de oxitocina, la función socioemocional cerebral y la morfometría en una muestra expuesta a trauma.

**Métodos:** Se incluyó personal policial holandés expuesto a trauma con (*n* = 38; 18 mujeres) y sin diagnóstico de TEPT (*n* = 40; 20 mujeres). Se evaluó la alexitimia mediante la Escala de Alexitimia de Toronto (TAS-20) por sus siglas en inglés. Se evaluó el nivel de oxitocina endógena salival durante el descanso empleando radioinmunoensayos. La reactividad de la amígdala y de la ínsula a estímulos socioemocionales se evaluaron mediante resonancia magnética funcional (RMf) y el volumen de la sustancia gris de la amígdala y de la ínsula se obtuvo empleando Freesurfer.

**Resultados:** La alexitimia fue mayor en pacientes con diagnóstico de TEPT en comparación con controles expuestos a trauma sin el diagnóstico (*F*(1,70) = 54.031, *p* < .001). Dentro del grupo de pacientes con diagnóstico de TEPT, la alexitimia estaba directamente asociada con la severidad del TEPT (*ρ*(36) = 0.497, *p* = .002). La alexitimia no estaba asociada con la exposición a trauma infantil (*β* = 0.076, *p* = .509), exposición a trauma relacionada con el trabajo policial (*β* = −0.107, *p* = .355), niveles de oxitocina (*β* = −0.164, *p* = .161), reactividad de la ínsula (*β* = −0.170, *p* = .158), o reactividad de la amígdala (*β* = −0.175, *p* = .135) o el volumen de la amígdala (*β* = 0.146, *p* = .209). El volumen de la ínsula estuvo directamente asociado a la alexitimia, aunque esta asociación no fue significativa luego de múltiples correcciones de pruebas. Los análisis bayesianos respaldaban la falta de asociación.

**Conclusiones:** No se observaron correlaciones neurobiológicas convincentes entre la alexitimia y los marcadores incluidos en este estudio. Sin embargo, este estudio confirmó niveles altos de alexitimia en pacientes con diagnóstico de TEPT, independientemente de la exposición a trauma, fundamentando la relevancia de la alexitimia en el fenotipo clínico del TEPT.

## Abstract (zh)

**背景：**述情障碍是一种无法识别自己情绪的症状，与创伤暴露和创伤后应激障碍（PTSD）有关。先前的研究表明催产素系统和社会情绪神经过程的参与。 然而，关于述情障碍的神经生物学研究很少，特别是在遭受创伤的人群中，值得进一步研究。

**目的：**探索创伤暴露样本中述情障碍、内源性催产素水平以及社会情绪脑功能和形态测量之间的关联。

**方法：**包括患有创伤后应激障碍（*n* = 38；18 名女性）和未患 PTSD（*n* = 40；20 名女性）的创伤暴露荷兰警察。使用多伦多述情障碍量表（TAS-20）评估述情障碍。 使用放射免疫测定法在休息时评估内源性唾液催产素。通过功能性 MRI 评估杏仁核和岛叶对社会情绪刺激的反应性，并使用 Freesurfer 得出杏仁核和岛叶灰质体积。

**结果：**与遭受创伤的对照组相比，PTSD 患者的述情障碍更高（*F*(1,70) = 54.031，*p* < .001）。 在 PTSD 患者中，述情障碍与 PTSD 严重程度呈正相关（ρ(36) = 0.497，*p* = .002）。 述情障碍与童年创伤暴露（*β* = 0.076，*p* = .509）、警察工作相关创伤暴露（*β* = −0.107，*p* = .355）、催产素水平（*β* = −0.164，*p* = .161）、脑岛（*β* = −0.170，*p* = .158）或杏仁核（*β* = −0.175，*p* = .135）反应性，或杏仁核体积（*β* = 0.146，*p* = .209）无关。岛叶体积与述情障碍呈正相关（*β* = 0.222，*p* = .016），尽管经过多次测试校正后并不显著。贝叶斯分析支持缺乏关联。

**结论：**当前研究中的任何标记均未观察到与述情障碍有令人信服的神经生物学相关性。 然而，当前的研究证实了 PTSD 患者的述情障碍水平很高，与创伤暴露无关，证实了述情障碍与 PTSD 临床表型的相关性。

## 1. Introduction

Alexithymia is a psychological construct associated with an inability to recognise and describe one’s own emotions. Individuals suffering from alexithymia often have intense affective states but lack the ability to identify or modulate these emotional states to the extent that the individual resorts to secondary dissociative states, compromising their self-awareness and perception of external reality (Zorzella et al., 2020). The construct of ‘alexithymia’, which literally means ‘no words for emotion’ (a = lack, lexis = word, thymos = emotion), is often characterised into four key facets: difficulty identifying feelings, difficulty describing feelings, externally oriented thinking, and diminished imagination/fantasy (Bräutigam & von Rad, 1977; Sifneos, 1973). Alexithymia is present in ∼10% of the population and is more prevalent in males (Eichhorn et al., 2014; Mattila et al., 2006; Salminen et al., 1999; Wright et al., 2018). Also, higher levels of alexithymia are observed in various psychiatric disorders compared to healthy controls, such as autism, depression, and posttraumatic stress disorder (PTSD; Frewen et al., 2008a; Kinnaird et al., 2019; Li et al., 2015; Taylor et al., 1997), and in relation to increasing levels of trauma-exposure, (Eichhorn et al., 2014; Terock et al., 2020; Zeitlin et al., 1993). Meta-analyses show alexithymia is seen in around 16% of PTSD patients and up to 43% in combat veterans with PTSD, and that alexithymia has been associated with increased PTSD severity (Frewen et al., 2008a; Putica et al., 2021). However, dose–response associations between alexithymia have also been observed in trauma-exposed individuals without PTSD, compared to non-trauma exposed individuals, suggesting that alexithymia is also associated with trauma independently of PTSD (Zeitlin et al., 1993). Taken together, alexithymia is a clinically relevant construct, particularly in relation to trauma and trauma-related psychopathology. Thus, a better understanding of alexithymia may improve our insight in vulnerability for psychiatric disorders.

Alexithymia has been described both as a personality trait (Messina et al., 2014; Taylor et al., 1997) and as a state that may be induced by (early) trauma exposure, as a repressive coping mechanism (Messina et al., 2014; Terock et al., 2020; Zeitlin et al., 1993). Some authors have suggested alexithymia develops as an integral manifestation of mental health disorders such as PTSD, and is a mere epiphenomenon related to emotional numbing symptoms (Badura, 2003; de Bruin et al., 2019; Yehuda et al., 1997). However, others have provided evidence that alexithymia and PTSD are distinct constructs, such that alexithymia is an inability to identify and communicate affective experiences, whereas emotional numbing in PTSD is a coping mechanism used to deal with negative affect (Eichhorn et al., 2014; Frewen et al., 2008b; Putica et al., 2021). Fitting with this latter view, longitudinal studies have suggested that alexithymia forms a predisposing risk factor for mental health disorders such as PTSD (Ledermann et al., 2020; McCaslin et al., 2006b; Tang et al., 2020). Clinically, high levels of alexithymia are associated with a diminished response to (trauma-focused) psychotherapy (Ogrodniczuk et al., 2011; Zorzella et al., 2020), which may be due to hypo-arousal and poorer habituation/fear extinction to emotional stimuli (Kosten et al., 1992; McCaslin et al., 2006a; Panayiotou & Constantinou, 2017; Taylor et al., 1997), or reduced therapeutic alliance and (perception of) social support (Nunes da Silva et al., 2018; Quirin et al., 2014). Thus, when investigating alexithymia, it is important to consider the possible interplay between trauma-exposure and PTSD.

The exact neurobiology of alexithymia remains largely unknown. However, the neuropeptide oxytocin might play a key role given its involvement in socio-emotional processing and emotion recognition (Grinevich & Neumann, 2021; Kendrick et al., 2018; Samur et al., 2013). Although studies of oxytocin and alexithymia are limited, alexithymia was associated with low levels of endogenous oxytocin in females with anorexia nervosa (Schmelkin et al., 2017). Interestingly, synthetic oxytocin administration may improve recognition of emotional expressions (Feeser et al., 2014), possibly most so for individuals scoring high on alexithymia, as observed by Luminet et al. (2011). Therefore, the association between oxytocin levels and alexithymia might have interesting therapeutic implications (Taylor & Bagby, 2013). Oxytocin administration might not only act on alexithymia symptoms directly by improving emotion recognition, but also by enhancing beneficial effects of social support and the communicative process of psychotherapy that are diminished with alexithymia (Nunes da Silva et al., 2018; Quirin et al., 2014).

Several studies have investigated brain function and structure in association with alexithymia in healthy populations. Previous research has suggested that brain regions involved in socio-emotional functioning, such as the amygdala and insula, may underlie alexithymia (Donges & Suslow, 2017; Moriguchi & Komaki, 2013; Van der Velde et al., 2013). The amygdala and insula are known for their role in processing emotion, whereby they direct visual attention toward emotional stimuli by calculating its emotional value and initiating emotional responses, as well as playing a role in interoception (Donges & Suslow, 2017; Uddin et al., 2017; Valdespino et al., 2017). Meta-analyses and reviews in healthy controls showed alexithymia was related to reduced amygdala and insula responses to negative stimuli, and reduced insula reactivity to positive socio-emotional stimuli (Donges & Suslow, 2017; Moriguchi & Komaki, 2013; Van der Velde et al., 2013). Also, more recent studies have related alexithymia to decreased amygdala reactivity to emotional faces and negative emotional stimuli (Goerlich, 2018; Morr et al., 2021), and to decreased insula reactivity to emotional stimuli in healthy individuals (Donges & Suslow, 2017; Taylor & Bagby, 2013). Also in PTSD patients, alexithymia was associated with decreased anterior insula reactivity to traumatic memory recall imagery (Frewen et al., 2006; 2008a). Interestingly, this association was not observed in trauma-exposed controls, suggesting stronger associations between alexithymia and insula reactivity particularly in PTSD populations (Frewen et al., 2006). In response to positive socio-emotional stimuli, in a sample of healthy participants, high levels of alexithymia were associated with decreased insula responses and increased hippocampal reactivity, specifically in individuals with high early life stress exposure (Aust et al., 2014a). This might suggest that also trauma-exposure, independent of PTSD diagnosis, may be related to stronger associations between alexithymia and insula & limbic reactivity.

A meta-analysis of structural brain studies also indicated a possible role for the insula and amygdala, as alexithymia was associated with smaller left insula and amygdala grey matter volume in analyses of over 2500 healthy individuals (Xu et al., 2018). Interestingly, in a large sample of brain-injury patients, insula damage was associated with increased levels of alexithymia, suggesting a potential causal role (Hogeveen et al., 2016). A limited number of studies investigating alexithymia and brain morphometry in trauma-exposed populations further suggested (early life) trauma exposure and PTSD status modulate the associations between alexithymia and brain structure. For example, early life stress was negatively associated with hippocampal volume only in high – and not low – alexithymic individuals (Aust et al., 2014b). Likewise, alexithymia was associated with medial prefrontal thickness in PTSD patients but not in non-trauma-exposed controls (Demers et al., 2015). As no trauma-exposed controls were included, this study could not distinguish between PTSD or trauma-related associations of alexithymia. Furthermore, these studies were relatively small and did not investigate insula or amygdala volumes, the region’s most consistently implicated in alexithymia (Xu et al., 2018).

Thus, although relevant neurobiological factors related to alexithymia have been identified in healthy populations, there is a paucity of neurobiological research on alexithymia in PTSD and trauma-exposed populations despite the repeatedly observed high levels of alexithymia in these groups. Alexithymia may not only increase the risk for developing trauma-related psychopathology (Ledermann et al., 2020; McCaslin et al., 2006b; Tang et al., 2020), but may also pose a possible hinderance on treating trauma-related disorders (Ogrodniczuk et al., 2011; Zorzella et al., 2020). More knowledge of the neurobiological mechanisms underlying alexithymia in trauma-exposed populations may provide explanations as to why this is the case. In particular first responders provide a relevant population, due to the high level of trauma they are subjected to, and the trauma-related disorders these individuals may develop as a result of their profession. Therefore, the primary aims of this study are to investigate the neurobiological correlates of alexithymia in a sample of both male and female trauma-exposed police officers with and without PTSD. As the current sample provided a rich variety of neurobiological measures, we have chosen to take a broad explorative approach, investigating several of the most promising makers in parallel. To this end, endogenous salivary oxytocin, and the structure and functional activity of the amygdala and insula will be investigated in relation to alexithymia. Based on previous research on the neurobiology of alexithymia, we expected alexithymia to be associated with reduced levels of endogenous oxytocin (Schmelkin et al., 2017). Furthermore, neuroimaging research in healthy populations suggests alexithymia is consistently associated with reduced amygdala responses, in particular in response to viewing negative emotional faces, indicating disturbed processing of these socio-emotional cues (Donges & Suslow, 2017; Goerlich, 2018; Van der Velde et al., 2013). As this has not yet been investigated in trauma-exposed individual or PTSD patients (Putica et al., 2021), we therefore investigated alexithymia associations with amygdala reactivity to negative emotional faces, a paradigm known for inducing robust amygdala responses (Hariri et al., 2002; Koch et al., 2016). Reduced insula responses have also been repeatedly shown in alexithymia to various socio-emotional tasks, in particular to positive emotional stimuli (Aust et al., 2014a; Donges & Suslow, 2017; Goerlich, 2018; Moriguchi & Komaki, 2013; Van der Velde et al., 2013). Therefore, we investigated insula responses to positive social stimuli in a social reward task, known to induce insula responses (Martins et al., 2021; Spreckelmeyer et al., 2009). Furthermore, based on a meta-analysis in healthy participants, we hypothesise smaller grey matter volumes in the insula and amygdala in association with alexithymia (Xu et al., 2018).

As secondary objectives, associations between alexithymia and PTSD symptoms, trauma exposure and sex will also be explored. Levels of trauma-exposure were expected to be high in both the control and PTSD participants, due to work-related trauma exposure. This is different from community-based PTSD case–control studies, where exposure levels are generally lower in control participants relative to PTSD patients. Therefore, this population of first responders represents an ideal group to disentangle associations of alexithymia with trauma-exposure and PTSD.

## 2. Methods

### 2.1. Sample and procedures

This study includes secondary analyses of data collected as part of a randomised controlled trial, which had the primary objective to investigate neural effects of a single intranasal oxytocin administration in trauma-exposed male and female police officers with and without PTSD (*n* = 80, Koch et al., 2016). Participants were recruited via advertisements (*n* = 65) and a psycho-trauma diagnostic outpatient clinic for police personnel (*n* = 15). Inclusion criteria included: aged between 18 and 65 years, and current or previous employment in the Dutch police force. Exclusion criteria included: daily use of psychotropic medication (e.g. antidepressants), use of systemic glucocorticoids, MRI contraindications, severe medical conditions, history of neurological disorders, colour blindness, and current pregnancy or breastfeeding. PTSD participants had to meet DSM-IV criteria for current PTSD, a Clinician-Administered PTSD Scale (CAPS) score ≧ 45 (Blake et al., 1995), and no other psychopathology except comorbid mild or moderate major depressive disorder (MDD) according to the Mini International Neuropsychiatric Interview when intake took place at the Amsterdam Medical Center (MINI; Sheehan et al., 1997; Van Vliet et al., 2000) or the Structured Clinical Interview for DSM-IV when intake took place at the outpatient clinic for police personnel (SCID; First et al., 2012; Van Groenestijn et al., 1999). Exposure to at least 1 traumatic event according to the DSM-IV PTSD A1 criterion and index trauma were assessed with the Life Events Checklist (LEC). The index trauma could be either private or work-related. For several police officers with PTSD, there was not one clear index trauma, but a series of traumatic events leading to the development of PTSD. PTSD patients with severe MDD (suicidal risk and/or psychotic symptoms), or current suicidal ideation were excluded. Trauma-exposed controls were matched to PTSD patients based on age, sex, education, and years of service, and had experienced at least 1 traumatic event according to the DSM-IV PTSD A1 criterion as assessed with the LEC, no current psychopathology (assessed with the MINI or SCID interview), and no current (subclinical)PTSD (assessed with the CAPS, CAPS score < 15; Blake et al., 1995), no lifetime PTSD and no current or lifetime MDD (assessed with the MINI or SCID interview).

The study was approved by the Institutional Review Board of the Academic Medical Center, University of Amsterdam, the Netherlands (protocol ID NL40122.018.12), registered in the Netherlands Trial Register (Registration ID NTR3516, https://trialregister.nl/trial/3368) and all participants provided oral and written informed consent prior to participation.

The current analyses were based on data obtained from a previous study (see Frijling et al., 2015; Koch et al., 2016; Nawijn et al., 2017). Measurements were obtained at several timepoints: An intake session (T1) including diagnostic interviews; a take-home self-report questionnaire that was completed between T1 and T2, which included alexithymia assessment; a first neuroimaging session (T2) which included endogenous salivary oxytocin assessment, structural brain imaging and functional brain imaging; and a second neuroimaging session (T3), which included functional brain imaging (see supplementary figure S1 for an overview of the study protocol). This study utilised a randomised cross-over design of intranasal placebo (saline, NaCl 0.9%) or oxytocin (40 IU Syntocinon) administration, in which half of the participants were randomised to receive placebo on T2 and oxytocin on T3, and the other half received oxytocin on T2 and placebo on T3. The salivary oxytocin measures taken at T2 were obtained prior to oxytocin or placebo administration. Structural neuroimaging data were acquired at the first scan session (T2). Immediate effects of oxytocin administration on brain structure were deemed unlikely and indeed, treatment condition (i.e. oxytocin vs. placebo) did not have a significant effect on brain structure (amygdala volume: *F*(1,71) = 0.308, *p* = .581; insula volume: *F*(1,71) = 0.532, *p* = .468, corrected for intracranial volume). The same functional neuroimaging tasks were performed at T2 and T3, and for this study, only functional neuroimaging data from the placebo sessions was included (either T2 or T3, depending on treatment order). Session order (i.e. placebo session being first or second fMRI session) had no effect on functional neuroimaging responses (amygdala response: *t*(69) = −1.061, *p* = .292; insula response: *t*(72) = −0.933, *p* = .354).

### 2.2. Measures

#### 2.2.1. Alexithymia

Alexithymia scores were based on the Twenty-Item Toronto Alexithymia Scale Questionnaire (TAS-20), a reliable and validated self-report measure of alexithymia, which has been shown to have strong concurrent and convergent validity (Bagby et al., 1994; Bagby et al., 2020). The TAS-20 demonstrated high internal consistency in the current sample (Cronbach’s alpha = 0.896). TAS-20 total scores were calculated as the sum of all 20 items (range 20–100), following Bagby et al. (1994). Missing items were mean-imputed, with maximum four missing items allowed for the total score. For sample descriptives, and to allow comparison with other samples, prevalence of alexithymia was calculated following guidelines by Bagby et al. (1994; scores ≤51 = no alexithymia, 52–60 = possible alexithymia, ≥ 61 = alexithymia).

#### 2.2.2. PTSD symptoms

The CAPS is a semi-structured diagnostic interview assessing the frequency and intensity of DSM-IV PTSD symptoms (Blake et al., 1995). Although DSM-IV and DSM-5 assessment of PTSD differs in several other ways (e.g. definition of trauma exposure criterion and three additional symptoms), these changes were found to have minimal impact on PTSD prevalence (American Psychiatric Association, 2013; Kilpatrick et al., 2013). A sum score of all 17 items was calculated (range 0–136). The CAPS scores demonstrated excellent internal consistency in the current sample (Cronbach’s alpha = 0.957). Furthermore, for exploratory analyses, CAPS subscales were calculated according to the four factor model (Asmundson et al., 2000; Friedman et al., 2011) by splitting the original DSM-IV cluster C, Avoidance and Numbing, into two separate clusters, Avoidance, and Emotional numbing. Symptom clusters were calculated by adding CAPS item scores in the following way; Intrusions (items B1-B5), Avoidance (C1-C2), Emotional numbing (C3-C7), and Arousal and reactivity (D1-D5).

#### 2.2.3. Psychotrauma exposure

The Early Trauma Inventory short form (ETI-SF), a reliable and valid self-report measurement of childhood trauma (Bremner et al., 2007), was used to assess the type and number of traumatic experiences before the age of 18. The ETI-SF demonstrated good internal consistency in the current sample (Cronbach’s alpha = 0.802). A sum score of all 21 items was calculated, as well as four subscales: General trauma (11 items, e.g. Exposure to Natural disaster, Serious accident, Serious injury/illness of parent, Separation of parents), Physical abuse (5 items, e.g. Slapped in the face, Punched or kicked, Pushed or shoved), emotional abuse (5 items, e.g. Often put down or ridiculed, Often ignored or made to feel you didn’t count; Most of the time treated in cold or uncaring way), and sexual abuse (5 items, e.g. Forced to touch intimate parts of someone else, Someone had genital sex against your will).

The Police Life Events Scale (PLES; Carlier & Gersons, 1992) is an occupational health-tool assessing the type and number of traumatic and stressful experiences related to police work, such as ‘Being shot’ or ‘Finding a dead body’. The total PLES score was calculated by summing the 41 items. The PLES demonstrated high internal consistency in the current sample (Cronbach’s alpha = 0.864).

#### 2.2.4. Endogenous salivary oxytocin

A single oxytocin saliva sample was taken from each participant at the start of the first scan session (T2), before intranasal administration and neuroimaging (for more details see Frijling et al., 2015, following Kagerbauer et al., 2013). Participants abstained from all food and drinks (except water), smoking, brushing teeth and exercise 1.5 h prior to saliva collection, and were instructed not to consume alcohol or drugs the night before their participation. After arrival, participants sat down comfortably in a quiet room and received instructions on the saliva collection procedure. Participants were asked to passively drool 4 ml of saliva into a tube, which was placed on ice and stored at −80°C. Due to MRI and participant scheduling constraints, collection times ranged from 09.30 to 19.00 h. Although endogenous salivary oxytocin levels might fluctuate across the day (Forsling, 2000), collection time did not correlate with salivary oxytocin levels in our sample (*r* = −0.072, *p* = .542). Samples were extracted prior to quantification. Oxytocin was quantified by a highly specific and sensitive radioimmunoassay using 0.8 ml of saliva (RIAgnosis, Munich, Germany), following current standards (Tabak et al., 2023).

#### 2.2.5. Neuroimaging acquisition

Neuroimaging took place at T2 and T3 on a 3T Philips Achieva MR system (Philips Medical Systems) with a 32-channel head coil. A high-resolution anatomical scan was acquired at T2 (see also Samples and procedures) with a FAST MPRage sequence (Field of view: 240 × 188 mm; voxel size: 1 mm^3^; repetition time: 8.2 s; echo-time: 3.8 ms; flip angle: 8°; 220 slices). Functional images were acquired at T2 and T3, using echo planar imaging sequences (Field of view: 240 × 240 mm; flip angle: 76; echo-time: 27.63 ms; repetition time: 2 s; voxel size: 3 mm^3^; acquisition matrix size: 80; 37 slices). Due to the crossover design of the current study, in which each participant was scanned twice (once after placebo administration and once after oxytocin administration), only functional neuroimaging data from the placebo sessions were used (see also Samples and procedures, and Supplementary figure S1).

#### 2.2.6. Structural neuroimaging

FreeSurfer version 5.3 was used to segment the neuroanatomical structures with the Desikan-Killiany Atlas (Martinos Center for Biomedical Imaging, Harvard-MIT, Boston, MA; http://surfer.nmr.mgh.harvard.edu/; Desikan et al., 2006; Wang et al., 2021). Structural MRI data was obtained during T2, assuming the administration of oxytocin would not have an acute effect on brain structure. Quality control was undertaken according to the ENIGMA consortium protocol (http://enigma.ini.usc.edu/protocols/imaging-protocols/). Left and right amygdala volume and left and right insula volume were summed to obtain bilateral amygdala and insula volumes (mm^3^).

#### 2.2.7. Emotional face matching task

The emotional face matching task (Hariri et al., 2002) consisted of three conditions: angry-fearful faces, happy-neutral faces, and a visuomotor control condition consisting of scrambled faces (for more details see Koch et al., 2016). Three stimuli were used in each trial – a cue at the top and two target stimuli below. Participants were asked to match a target with the cue based on emotional expression (emotional faces) or orientation (scrambled faces). Imaging data were analysed using SPM8 (http://fil.ion.ucl.ac.uk/spm/software/spm8). Individual estimates of amygdala reactivity to emotional faces were extracted from 5 mm spheres surrounding peak task activation in the left and right amygdala (MNI xyz = −20, −8, −16; xyz = 24, −10, −14, *p* < .05, family-wise-error (FWE) corrected) under placebo for the contrast ‘fearful-angry’ versus ‘happy-neutral’ faces, and averaged across hemispheres (Koch et al., 2016; Van Zuiden et al., 2019). This contrast aims to measure amygdala responses to negative socio-emotional stimuli, relative to neutral and positive stimuli.

#### 2.2.8. Social incentive delay task

The social incentive delay task (SID; Spreckelmeyer et al., 2009) is a reaction time task in which participants are rewarded with a happy face or punished with an angry face (for more details see Nawijn et al., 2017). The task consisted of reward, punishment, and neutral trials. In reward trials, hits resulted in social reward (i.e. happy face), misses in neutral feedback (i.e. scrambled face); in punishment trials, hits resulted in neutral feedback, misses in social punishment (i.e. angry face); in neutral trials, hits and misses resulted in neutral feedback. For the purpose of the current study, as we were interested in responses to positive feedback in the form of social stimuli, thus only reward feedback trials from the placebo session were included. Task difficulty was tailored to individual performance. Imaging data were analysed with SPM8. As task effects were strongest in the left insula, and in the right insula no activity was observed reaching the significance threshold (*p* < .05, FWE-corrected, Nawijn et al., 2017), only the left insula was used. Thus, insula reactivity to social reward was extracted from a 5 mm sphere around the peak activation in the left anterior insula (MNI xyz = −36, 4, 10, *p* < .05, FWE-corrected) under placebo for the contrast ‘social reward feedback’ versus ‘neutral reward feedback’. This contrast aims to measure insula responses to positive social reward feedback (i.e. viewing happy faces in the context of a performance-based reward task), relative to neutral non-social reward feedback.

### 2.3. Statistical analyses

Statistical analyses were performed using R version 4 (R Core Team, Vienna, Austria, 2021; https://www.R-project.org/), R Studio version 22 (RStudio team, Boston, MA, USA, 2020; http://www.rstudio.com/) and SPSS version 28 (IBM Corp., Armonk, NY, USA, 2021). Alexithymia scores had acceptable levels of skewness and kurtosis (skewness = 0.516, kurtosis = −0.763) and no outliers were observed, i.e. no participants scoring >3SD from the mean. Within PTSD patients, CAPS scores also approached normal distribution (skewness = 0.280, kurtosis = −0.509) and no outliers were observed. Childhood trauma scores and salivary oxytocin levels showed high levels of skewness and kurtosis (childhood trauma: skewness = 1.61, kurtosis = 2.674; oxytocin levels skewness = 1.962, kurtosis = 2.989), and were log-transformed to approach a normal distribution (childhood trauma log-transformed skewness = −0.084, kurtosis = −0.387; oxytocin log-transformed skewness = 0.873, kurtosis = −0.174). Childhood trauma subscales were analysed using non-parametric methods. All other variables showed normal levels of skewness and kurtosis (policework-related trauma exposure skewness = −0.225, kurtosis = −0.459; bilateral amygdala volume skewness = 0.119, kurtosis = −0.172; bilateral insula volume skewness = 0.571, kurtosis = 1.376; amygdala responses to emotional faces skewness = 0.208, kurtosis = 1.323; insula responses to social reward skewness = −0.197, kurtosis = 0.610),

As initial exploratory analyses, a two-way ANOVA was used to measure the effects of PTSD diagnosis, sex and PTSD-by-sex interaction on alexithymia. Associations between alexithymia, PTSD symptom severity, and PTSD symptom subscales Intrusions, Avoidance, Emotional numbing, and Arousal and reactivity, were investigated within PTSD patients only, using Spearman’s rho (ρ) correlation coefficients. To assess if associations between alexithymia and PTSD symptoms were dependent on trauma-exposure, additional partial correlations were performed while correcting for childhood trauma (ETI-SF) and work-related trauma exposure (PLES).

Associations between alexithymia, childhood trauma (ETI-SF) and work-related trauma (PLES) were investigated with separate hierarchical linear regression analyses. In this stepped modelling approach, the first models included one of the trauma-exposure variables as the outcome (i.e. childhood trauma or work-related trauma), and alexithymia score as the predictor variable. Next, stepwise regression was performed to test if potential confounders should be included in the model. Sex, age, education (i.e. self-reported highest achieved educational degree was re-coded to low, middle or high education level following the Netherlands Central Bureau of Statistics guidelines (CBS, 2016)), and years of service (for work-related trauma models only) were considered as potential confounders based on literature (e.g. Benjet et al., 2016; Mattila et al., 2006). Confounders were included in the model as covariate if they contributed significantly to the outcome variable (*p* < .05) using stepwise regression. In a second step, PTSD diagnosis was included to investigate if associations with alexithymia were dependent on PTSD diagnosis. Potential associations between alexithymia or trauma exposure subtypes were explored with Spearman’s ρ correlation analyses including childhood trauma subscales General trauma, Physical abuse, Emotional abuse and Sexual abuse.

The primary research questions, investigating the relation between alexithymia and salivary oxytocin, amygdala and insula reactivity and volume, were assessed with separate hierarchical linear regression analyses. The first models included one of the neurobiological variables as the outcome (i.e. salivary oxytocin, amygdala reactivity, insula reactivity, amygdala volume, or insula volume), and alexithymia score as the predictor variable. Next, stepwise regression was performed to test if potential confounders should be included in the model. Sex, age and education were considered as potential confounders for all outcome variables (e.g. Mattila et al., 2006) and included in the models if they contributed significantly to the outcome variable (*p* < .05) using stepwise regression. For oxytocin models, we additionally considered self-reported hormonal contraception and self-reported menopause state as covariates, following Frijling et al. (2015), as oral contraceptive use and circulating estrogen and progesterone levels might influence oxytocin levels (e.g. Cyranowski et al., 2008; Salonia et al., 2005). Intracranial volume (ICV) was considered as a potential additional covariate for amygdala and insula volume models using stepwise regression, to correct for head size following standard practices in structural brain imaging (Barnes et al., 2010). As post-hoc analyses, neuroimaging variables were also analysed per hemisphere, to check for potential unilateral effects. Additionally, all models were run including PTSD diagnosis as a covariate to investigate if associations with alexithymia were dependent on PTSD diagnosis. *p*-Values were corrected for multiple testing using false discovery rate (FDR; Benjamini & Hochberg, 1995), FDR-corrected *p*-values<.05 were considered significant.

Bayesian statistical testing was utilised to identify the probability of the null and alternative hypotheses given the observed data, expressed as a Bayes Factor (BF; Kruschke & Liddell, 2018). The full versions of the hierarchical linear regression models for each of the neurobiological outcome variables (salivary oxytocin, amygdala reactivity, insula reactivity, amygdala or insula volume), using alexithymia and relevant covariates as predictors (alternative hypothesis), were compared to the same models without alexithymia (null hypothesis). BF values >1 were interpreted to support the alternative hypothesis (between 3–10 = moderate, 10–30 = strong, and >30 = very strong evidence), and values <1 supporting the null hypothesis (Kruschke & Liddell, 2018).

## 3. Results

### 3.1. Sample description

Alexithymia TAS-20 scores were available for 78 participants, of which 38 with PTSD (18 females) and 40 healthy controls (20 females). For descriptive statistics, see Table 1. Total alexithymia scores ranged from 24–79 (mean 49.23, SD 13.77). See Supplementary Figure S2 for histograms of alexithymia scores.

**Table 1.** Sample descriptives.

*Demographics*	
Age, mean (SD), (range)	40.06 (9.72), (22–59)
Sex, *n* (%)	
Males	40 (51%)
Females	38 (49%)
Use of hormonal contraceptives, *n* (% of females)	17 (45%)
Menopausal, *n* (% of females)	4 (11%)
Education	
Low	0 (0%)
Middle	67 (86%)
High	11 (14%)
Years of police service, mean (SD), (range)	16.50 (9.95), (4–40)
*Clinical & psychotrauma characteristics*	
PTSD diagnosis, *n* (%)	38 (49%)
Comorbid Major Depressive Disorder, *n* (% of PTSD patients)	9 (24%)
PTSD symptom severity (CAPS total score), mean (SD)	35.71 (33.77)
Police work-related trauma (PLES), mean (SD)	19.06 (7.86)
Childhood trauma (ETI-SF), median (IQR)	4.00 (1.75–6.25)
*Alexithymia*	
Alexithymia (TAS-20 total score)	49.23 (13.77)
Alexithymia level	
No alexithymia	49 (63%)
Possible alexithymia	10 (13%)
Alexithymia	19 (24%)
*Neurobiological characteristics*	
Salivary oxytocin, median (IQR)	1.00 (0.30–3.00)
Left amygdala reactivity, mean (SD)	0.187 (0.43)
Right amygdala reactivity, mean (SD)	0.169 (0.31)
Left insula reactivity, mean (SD)	−0.047 (0.86)
Amygdala volume (bilateral mm^3^), mean (SD)	3303.35 (358.32)
Insula volume (bilateral mm^3^), mean (SD)	14173.72 (1426.37)
Intracranial volume (mm^3^), mean (SD)	1335701.36 (223347.48)

### 3.2. Alexithymia and PTSD

Individuals with a PTSD diagnosis had significantly higher alexithymia scores than trauma-exposed controls (*F*(1,74) = 56.655, *p* = <.001). No difference was observed in alexithymia scores between males and females (*F*(1,74) = 0.061, *p* = .806) and there was no significant interaction between PTSD diagnosis and sex (*F*(1,74) = 0.017, *p* = .895). Alexithymia score distributions per group are illustrated in Supplementary Figure S3.

**Figure 1.**
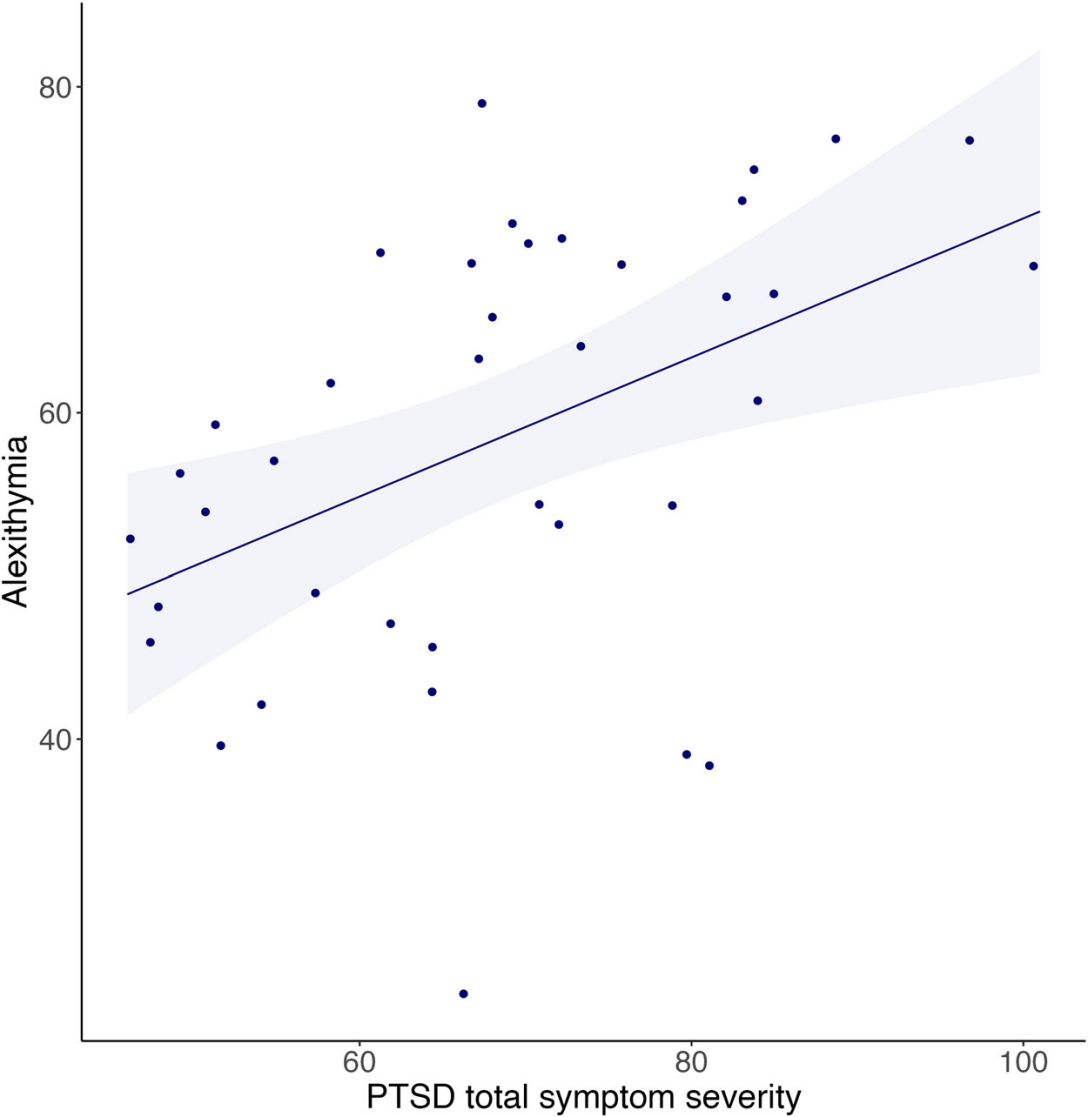
Scatter plot of linear associations between alexithymia (TAS-20 total score) & PTSD total symptom severity score (CAPS total score; *r*(36) = 0.457, *p* = .004), within PTSD participants only (*n* = 38). Plot includes a linear regression line and 95% confidence intervals (light blue band).

Within PTSD patients, alexithymia scores were significantly positively correlated with PTSD symptom severity (CAPS total scores; *ρ*(36) = 0.497, *p* = .002, *n* = 38, see Figure 1). The association between alexithymia and PTSD symptoms remained of similar strength and statistical significance when correcting for childhood and work-related trauma exposure (partial correlation Alexithymia – CAPS total score: *ρ*(33) = 0.519, *p* = .001), and when excluding one potentially influential case (TAS-20 score = 24, CAPS total score = 66, Cook’s distance = 0.218) (correlation Alexithymia – CAPS total score: *ρ*(35) = 0.497, *p* = .002).

For exploratory analyses of PTSD symptom subscales and total alexithymia scores, see Supplementary Table S1 and Figure S4.

### 3.3. Alexithymia and psychotrauma exposure

**Figure 2.**
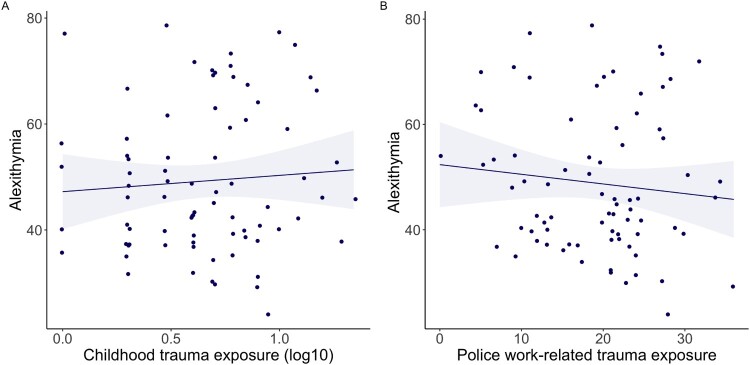
Scatterplots of linear associations between Alexithymia, Childhood trauma, and Police work-related trauma exposure, including linear regression line and 95% confidence intervals. Panels display the association between Alexithymia (TAS-20 total score) and A. Childhood trauma exposure (ETI-SF total score, log10 transformed), which was not significantly associated with alexithymia levels (*β* = 0.076, *p* = .509, *n* = 78) and B. Police work-related trauma exposure (PLES total score), which was not significantly associated with alexithymia levels (*β* = −0.068, *p* = .490, *n* = 77).

Alexithymia levels were not significantly associated with total childhood trauma exposure (*β* = 0.076, *p* = .509*, n* = 78, Figure 2(A), Supplementary Table S2A). Sex, age and education did not add to the model (*p* > .05) and were therefore not included. None of the childhood trauma subtypes were associated with alexithymia, as explored with correlation analyses (general trauma, *ρ*(76) = 0.022, *p* = .847; physical abuse, *ρ*(76) = 0.048, *p* = .674; emotional abuse, *ρ*(76) = 0.218, *p* = .056; sexual abuse, *ρ*(76) = −0.042, *p* = .717). Alexithymia was not significantly associated with Police work-related trauma exposure (PLES total score; *β* = −0.068, *p* = .490, *n* = 77, Figure 2(B), Supplementary Table S2B). Sex (*β* = −0.353, *p* < .001), age (*β* = −0.470, *p* = .041) and years of service (*β* = 0.832, *p* < .001) did contribute to work-related trauma exposure and were therefore included in the model, whereas education did not add to the model (*p* > .05) and was not included. Including these covariates did not affect the association between alexithymia and work-related trauma exposure (*β* = −0.117, *p* = .368). For a correlation matrix of all alexithymia and trauma exposure (sub)scales, see Supplementary Table S3A and S3B.

### 3.4. Alexithymia and endogenous salivary oxytocin levels

**Figure 3.**
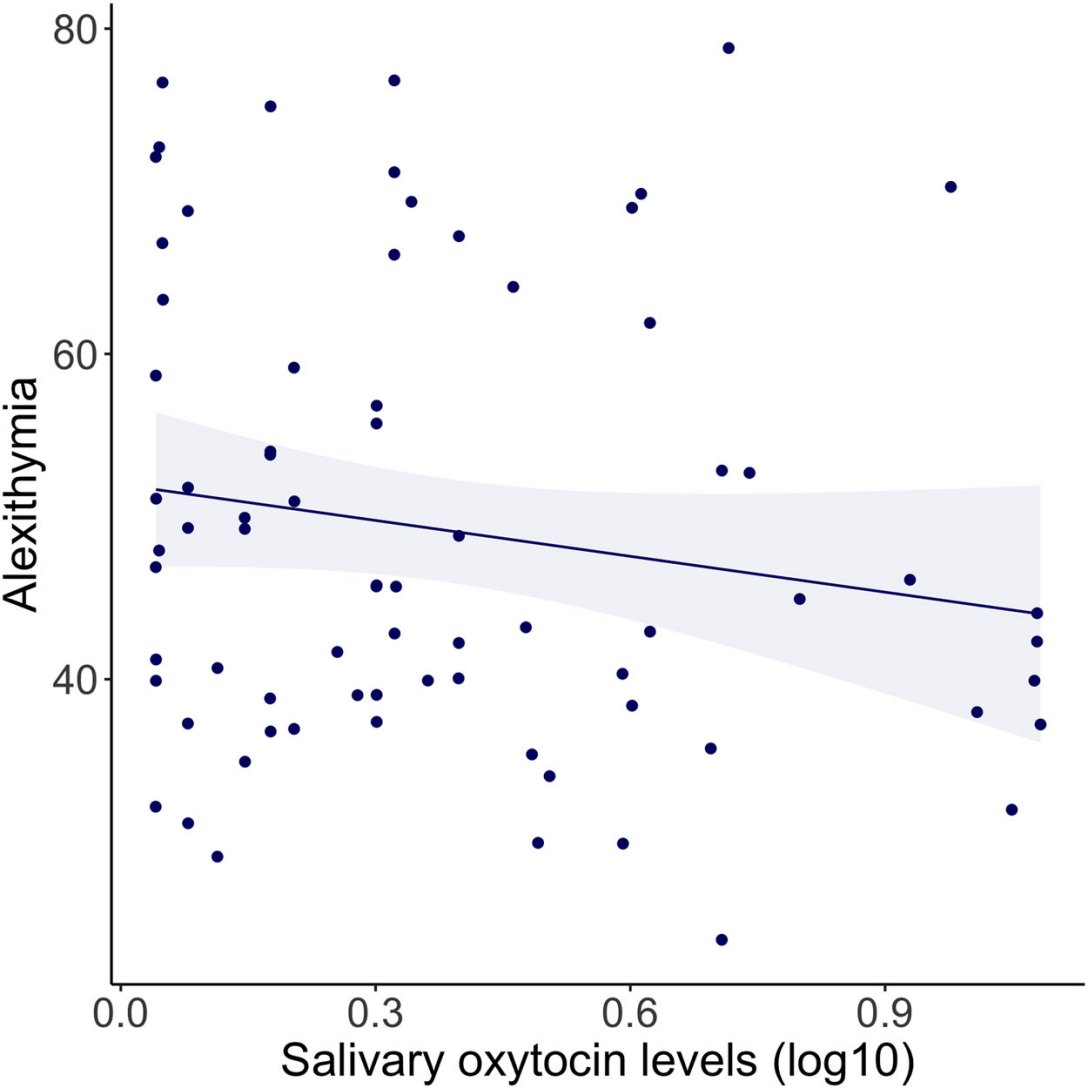
Scatterplot of linear association between alexithymia (TAS-20 total scores) and endogenous salivary oxytocin levels (pg/ml, log10 transformed), including linear regression line and 95% confidence intervals. Alexithymia was not significantly associated with endogenous salivary oxytocin levels (*β* = −0.164, *p* = .161, BF = 0.566±0%, *n* = 75).

Alexithymia was not associated with endogenous salivary oxytocin levels (*β* = −0.164, *p* = .161, pFDR = 0.201, BF = 0.566+−0%, *n* = 75), see Figure 3 and Supplementary Table S4. These associations did not change when also including PTSD in the model. Sex, education, hormonal contraceptives use or menopause status did not add to the model (*p* > .05) and were therefore not included.

### 3.5. Alexithymia and amygdala and insula reactivity to socio-emotional stimuli

**Figure 4.**
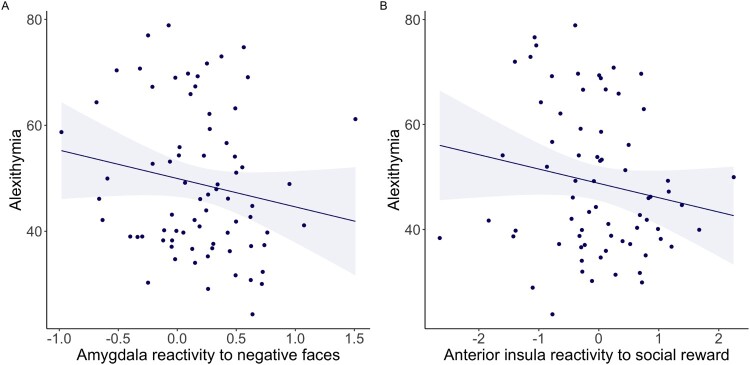
Scatterplots of alexithymia (TAS-20 total scores) and neural reactivity to socio-emotional stimuli, including linear regression line and 95% confidence intervals. A. Bilateral amygdala reactivity to negative relative to positive/neutral faces (arbitrary units), which was not significantly associated with alexithymia (*β* = −0.175, *p* = .135, BF = 0.595±0%, *n* = 74) and B. Left anterior insula reactivity to social reward relative to neutral reward (arbitrary units), which was not significantly associated with alexithymia (*β* = −0.170, *p* = .158, BF = 0.584±0%, *n* = 71).

Alexithymia was not significantly associated with bilateral amygdala reactivity to negative emotional faces (*β* = −0.175, *p* = .135, pFDR = 0.201, BF = 0.639+−0%, *n* = 74), nor with left anterior insula reactivity to social reward (*β* = −0.170, *p* = .158, pFDR = 0.201, BF = 0.584±0%, *n* = 71), see Figure 4 and Supplementary Table S5. These associations did not change when also including PTSD in the model. Sex and education did not add to the models (*p* > .05) and were therefore not included as covariates. Post-hoc tests exploring unilateral amygdala reactivity showed that alexithymia was not associated with left (*β* = 0.169, *p* = .150) nor right amygdala reactivity to emotional faces (*β* = 0.142, *p* = .228). Right insula reactivity to social reward was not investigated as there was no activation in this region (see paragraph 2.3.9).

### 3.6. Alexithymia, amygdala and insula volume

**Figure 5.**
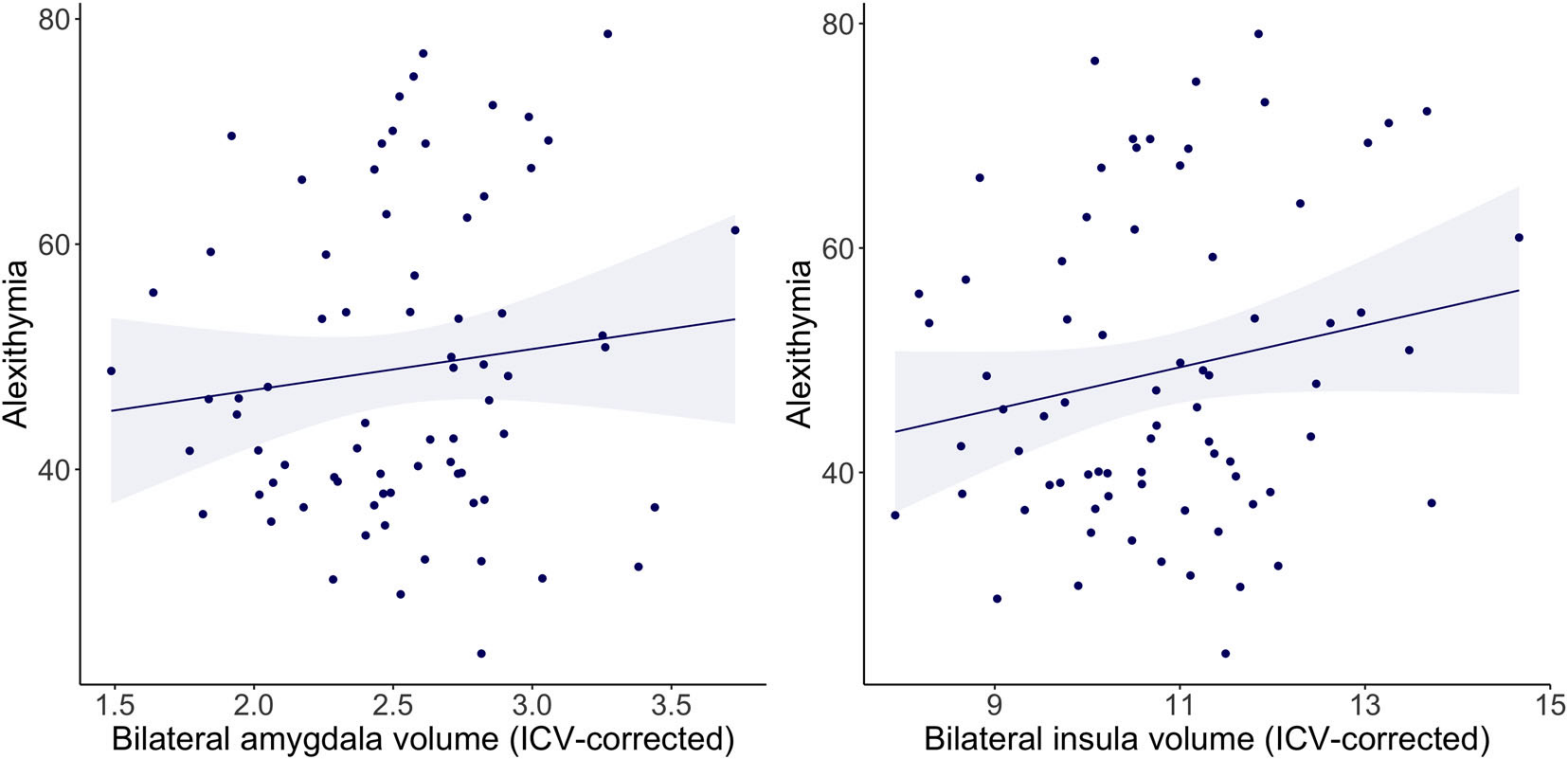
Scatter plots of linear associations between alexithymia (TAS-20 total scores) and amygdala and insula volume, including linear regression line and 95% confidence intervals. A. bilateral amygdala, which was not significantly associated with alexithymia (*β* = 0.146, *p* = .209, BF = 0.64±0%, *n* = 74) and B. bilateral insula volume, which was positively associated with alexithymia albeit subthreshold after FDR-correction (*β* = 0.222, *p* = .016, pFDR = 0.080, BF = 2.76±0.01%, *n* = 74). Amygdala and insula volume are corrected for intracranial volume (amygdala volume mm^3^ / ICV mm^3^ * 1000; insula volume mm^3^ / ICV mm^3^ * 1000).

Alexithymia levels were not associated with bilateral amygdala volume (*β* = 0.146, *p* = .209, BF = 0.64±0%, *n* = 74, Figure 5(A), Supplementary Table S6). These associations did not change when also including PTSD in the model. Post-hoc tests exploring unilateral amygdala volumes showed that alexithymia was not associated with left (*β* = 0.243, *p* = .014) nor right amygdala volume (*β* = 0.182, *p* = .045). Alexithymia was positively associated with bilateral insula volume (*β* = 0.222, *p* = .016, BF = 2.76±0.01%, *n* = 74; Figure 5(B), Supplementary Table S6), although this remained subthreshold after FDR correction for multiple testing (pFDR = 0.080). Associations were stronger when additionally including PTSD diagnosis in the model (*β* = 0.364, *p* = .003, BF = 2.06±1.00%). Splitting the participants by group suggested that a positive association between alexithymia and bilateral insula volume was present in both control participants (*β* = 0.346, *p* = .009), and PTSD patients (*β* = 0.272, *p* = .037). Post-hoc tests exploring unilateral insula volumes showed that associations with alexithymia were present for both left (*β* = 0.243, *p* = .014) and right insula (*β* = 0.182, *p* = .045). Intracranial volume was included as a covariate as it contributed significantly to the model for insula volume (*β* = 0.623, *p* < .001), whereas sex and education did not add to the models (*p* > .05) and were therefore not included.

## 4. Discussion

This study investigated the neurobiological correlates of alexithymia in a sample of male and female trauma-exposed police officers with and without PTSD, aiming to contribute to the understanding of alexithymia. To this end, endogenous salivary oxytocin, and the structure and functional activity of the amygdala and insula were investigated in relation to alexithymia, as well as associations with PTSD, trauma exposure, and sex. In line with previous meta-analyses (Frewen et al., 2008a; Putica et al., 2021), PTSD symptom severity was strongly positively associated with alexithymia total score. Moreover, alexithymia correlated specifically with the PTSD symptom clusters Emotional numbing, and Arousal, but not with the Intrusions, or Avoidance symptom clusters. Males and females did not differ in alexithymia levels. Also, we did not observe any associations between alexithymia and childhood or work-related trauma-exposure. The hypothesised associations between alexithymia and endogenous salivary oxytocin, and the structure and functional activity of the amygdala and insula and alexithymia were not observed in the current sample, confirmed by Bayesian statistical testing. Although initial investigations showed a weak positive relationship between alexithymia and bilateral insula grey matter volume, this was no longer significant after correcting for multiple comparisons. The Bayesian analyses showed evidence supporting the absence of associations with alexithymia for all variables with the exception of insula volume with a BF ∼ 2.76, supplying weak evidence for a positive association with alexithymia.

The strong association between PTSD and alexithymia replicates previous meta-analytic findings, where PTSD patients score higher on alexithymia than trauma-exposed controls (Frewen et al., 2008a; Putica et al., 2021). Contrary to expectations (Mattila et al., 2006; Salminen et al., 1999; Wright et al., 2018), no sex differences were observed in alexithymia scores. Potentially, the selective sample of male and female police officers may have played a role here, as previous studies suggested police work is associated with a specific ‘police personality’ profile in both males and females (e.g. Gould & Volbrecht, 1999; Grubb et al., 2015), hence (female) first responders may form a unique sample that might explain inconsistencies relative to previous studies. This may limit the generalizability of these findings to other trauma exposed populations. Interestingly, associations between PTSD symptoms and alexithymia were independent of trauma exposure, and alexithymia was not associated with childhood or work-related trauma exposure in our highly trauma-exposed sample. This concurs with previous observations that alexithymia was associated with PTSD but not with trauma exposure (e.g. Badura, 2003; Spitzer et al., 2007; Yehuda et al., 1997). Possibly, alexithymia is either a pre-existing risk factor for PTSD (McCaslin et al., 2006b; Tang et al., 2020), or it develops in parallel with or in response to PTSD. For example, alexithymia may be a repressive coping mechanism allowing individuals to deal with the emotional distress associated with PTSD (de Bruin et al., 2019; Güleç et al., 2013; Messina et al., 2014; Wojciechowska et al., 2021). However, the lack of associations between alexithymia and childhood and adult trauma are in contrast with previous studies, which again, may be due to the limited generalizability of this selective sample of first-responders (Terock et al., 2020; Zeitlin et al., 1993). Also, specifically in first responders, such as police officers and emergency call operators, positive associations between alexithymia and work-related trauma exposure have been observed (McCaslin et al., 2006b; Wojciechowska et al., 2021). Furthermore, alexithymia might be most strongly associated with emotional neglect in childhood (Frewen et al., 2008a, Kahn & Jaffee, 2022; Terock et al., 2020), which was only partially covered in our measure of childhood trauma (emotional neglect items were combined with emotional abuse items in the emotional abuse subscale). In future research, it would be relevant to further investigate emotional neglect, to gain a more complete view of the associations between alexithymia and childhood adversity. Furthermore, the control group in the current study, with high levels of trauma-exposure and absence of psychopathology, may have represented a highly resilient sample, which could also explain differences with other (community-based) samples. It would be interesting to achieve an accurate understanding of the temporal order of alexithymia and PTSD while considering (childhood) trauma exposure, for example by longitudinally following individuals in high trauma-exposure professions, including measurements before commencement of the profession.

Contrary to expectations (Schmelkin et al., 2017), there was a lack of association between endogenous salivary oxytocin and alexithymia. This may be due to how the oxytocin was measured (single salivary sample under resting conditions). For one, basal salivary levels may not fully reflect levels of oxytocin in the brain (Valstad et al., 2017). Secondly, oxytocin levels may vary within individuals across days, suggesting that repeated sampling across several days may provide a better reflection of average basal oxytocin levels (Martins et al., 2020). Alternatively, deficits in emotion recognition may be particularly reflected in endogenous oxytocin release in response to social stimuli (Matsunaga et al., 2020), although basal oxytocin levels in plasma have also been associated with emotional functioning (Spilka et al., 2022).

Overall, this study found no significant convincing associations between alexithymia and the structure and functional activity of the amygdala and insula. This contrasts with most existing literature in healthy populations, with a meta-analysis showing smaller insula and amygdala volumes in relation to alexithymia (Xu et al., 2018) and decreased activation of the insula and amygdala towards socio-emotional stimuli in association with alexithymia (Aust et al., 2014a; Donges & Suslow, 2017; Goerlich, 2018; Taylor & Bagby, 2013; Van der Velde et al., 2013). One possible explanation is that alexithymia displays stronger associations with neural correlates in healthy populations relative to the current sample of trauma-exposed police officers with and without PTSD. Although there was a wide range of alexithymia levels in the current sample, and we expected neurobiological measures to be stronger associated with alexithymia in trauma-exposed and PTSD populations (e.g. Aust et al., 2014a; 2014b; Demers et al., 2015; Frewen et al., 2006), high levels of trauma-exposure and (resilience to) PTSD may have introduced additional noise relative to healthy participants. Thus, the findings of this study may be specific to first responders and not generalise to community-based populations. Another reason for this lack of associations may be that the neural correlates of alexithymia lie within specific subregions of the amygdala and insula, or in other brain regions that we have not investigated. For example, the meta-analyses on structural brain correlates of alexithymia observed smaller regional volumes primarily within the left anterior insula and left amygdala, using voxel-based morphometry. Within the current study, we may have missed more specific regional associations such as within one hemisphere or within the anterior insula, as we investigated total bilateral insula and amygdala volume based on freesurfer-derived regional segmentations. Also, the functional imaging tasks show methodological differences with some previous studies. For example, functional imaging tasks designed to probe recognition of the participants’ own emotions may have been more sensitive to alexithymia-related deficits, although reduced amygdala and insula reactivity have been observed in response to emotional faces paradigms and positive social stimuli similar to the current study (Aust et al., 2014a; Donges & Suslow, 2017; Van der Velde et al., 2013). The amygdala is known to show fast habituation, thus there might also have been differences in temporal dynamics that have not been investigated in this study. For example, it has been found that using magneto-encephalography (MEG) to identify temporal dynamics in the brains of combat veterans with and without PTSD, differences in amygdala activity in response to emotional faces were observed as early as 50 ms (Badura-Brack et al., 2018). Thus, this may also be relevant to investigate in relation to alexithymia in future research. On the other end of the spectrum, instead of early emotional processing in the amygdala and insula, alexithymia may also be related to neural deficits in higher-order processing and conscious awareness of emotions, for example in the anterior cingulate and medial prefrontal cortex which were not included as regions of interest in the current study (Lane et al., 1997; Morr et al., 2021; Van der Velde et al., 2013). This notion is not new to alexithymia research, as the ‘blindfeel’ hypothesis posed by Lane et al. (1997) states that ‘alexithymia consists of deficient development of conscious awareness of emotion’, suggesting that individuals with alexithymia do experience emotion, but lack the ability to consciously identify the experiences as emotion. This ties in with the findings of this study that there is a positive association between alexithymia and the arousal symptoms subscale of PTSD (see supplement), such that an alexithymic individual still experiences arousal in response to emotional stimuli but might not associate or recognise the arousal in union with a particular emotion. Alternatively, deficits may arise due to dysfunction in neural communication networks such as the default mode and salience network, rather than regional reactivity (Colic et al., 2016; Liemburg et al., 2012; Putica et al., 2021). Hence, further research into conscious processing of (one’s own) emotions and functional connectivity networks in the brain may be worthwhile.

The tentatively positive association between alexithymia and insula grey matter volume observed in this study suggests that the insula is larger in individuals with higher alexithymia scores, and highlights the need for investigation in a larger sample. This is contrary to our expectations based on a meta-analysis in healthy participants (Xu et al., 2018), although several single studies have previously noted larger insula volumes as well, indicating that there is some variability in these observations (Farah et al., 2018; Goerlich-Dobre et al., 2014; Zhang et al., 2011). Anterior insula volume has been positively correlated with expressive repression (Giuliani et al., 2011), that is, enhanced suppression of emotions after an emotional response has been generated, again in line with the blindfeel hypothesis by Lane et al. (1997). On this notion, the insula’s role in emotional awareness and interoception might propose that an increased volume accommodates increased neural communication regarding interoceptive processes, which would contradict previous findings related to *decreased* interoceptive processes and insula reactivity (Ernst et al., 2014; Morr et al., 2021; Uddin et al., 2017; Valdespino et al., 2017; Van der Velde et al., 2013). Alternatively, the increased somatisation complaints seen in alexithymia (Fares et al., 2019; Taylor et al., 1997) may also explain a larger insula volume. While there is a weak indication from the Bayesian analysis that there is a true association between the bilateral insula volume and alexithymia, this relationship is contrary to the expectations of this study based on previous research that a *smaller* insula volume would be related to alexithymia (Xu et al., 2018), and caution is warranted in the interpretation of these findings.

### 4.1. Strengths and limitations

A limitation of the current study is the small sample size. Although with a sample of 78 participants it was sufficiently powered to detect associations of medium-to-large effect size (e.g. as based on Schmelkin et al., 2017; Xu et al., 2018), this resulted in low power to identify associations of small effect size. Overall, associations between alexithymia and salivary oxytocin, and amygdala and insula reactivity were in the expected direction, but too small to be of clinical or statistical significance. Bayesian testing further supported an absence of the hypothesised associations.

Some limitations must be noted regarding the included measurements in the current study. Firstly, PTSD diagnosis and symptoms were assessed using the DSM-IV-based CAPS interview (Blake et al., 1995), which limits the comparability of our findings to DSM-5 based PTSD symptoms. Moreover, limited information was available regarding the index trauma (i.e. type of event, private or work-related, time since the trauma). This restricted the investigation of potential effects of index trauma type and time since trauma. Also, while the alexithymia TAS-20 scale is a validated standard measure in alexithymia research, it is a single self-report measure and therefore might lack the ability to encompass the construct in its entirety. The field might benefit from further research and consensus particularly concerning the externally oriented thinking and fantasy elements of alexithymia, as well as better validation in clinical samples (e.g. Bagby et al., 2020; Kooiman et al., 2002). However, a large body of research has shown the TAS-20’s reliability and validity (Bagby et al., 2020), suggesting its capacity to identify alexithymia is warranted. Secondly, the current data were part of a larger RCT investigating the effects of a single administration of intranasal oxytocin. It is important to note that the larger RCT was not primarily designed to investigate alexithymia, and the current study included secondary analyses on this dataset. However, while the study design may not have been optimal, the sample portrayed a wide distribution of alexithymia scores, warranting the investigations undertaken in this study. Furthermore, as all functional measures were taken either prior to oxytocin administration or more than seven days after oxytocin administration, we believe there are no direct effects of oxytocin on the described outcome measures. Indeed, there were no significant differences in amygdala or insula volume between participants receiving placebo and those receiving oxytocin immediately before structural scanning. For functional neuroimaging, only placebo session data was used. However, as the order of oxytocin and placebo sessions was counterbalanced, we also confirmed that session order had no significant effect on our functional outcomes of interest (amygdala faces; insula reactivity to social reward, see methods section 2.1). Taken together, the effects are likely limited, however, the current set-up might have influenced our findings relative to other alexithymia studies. Furthermore, concerning the salivary oxytocin assessment, recent recommendations for measuring trait-like levels of basal endogenous oxytocin suggest to include several measurements at different days and take the average to increase reliability (Martins et al., 2020; Tabak et al., 2023). In the current study only one sample was taken. This might limit the interpretation of our salivary oxytocin measurements. As a result, we can only draw conclusions about short-term or state-like levels of endogenous salivary oxytocin at the day of measurement, as these levels are known to fluctuate within individuals across days (Martins et al., 2020). Yet, as within-session reliability for oxytocin measurements is high (Martins et al., 2020), the sample taken should confer a reliable measure of salivary oxytocin levels at that time. Furthermore, it must be noted that in the current neuroimaging approach, a more general functioning of the amygdala and insula was investigated across (specific elements of) different neuroimaging paradigms, and that this is by no means an exhaustive investigation of neurobiological functioning. Although this allowed us to explore several promising neurobiological correlates within a single study, the design should be considered exploratory, rather than an in-depth investigation. This set-up allows us to draw conclusions only concerning these specific elements (i.e. overall bilateral amygdala and insula volume, bilateral amygdala response to fearful and angry faces, and left insula responses towards social reward feedback). Future studies may perform more in-depth investigations of neural correlates of alexithymia, e.g. investigating other brain regions or taking voxel-based approaches, distinguishing between specific types of emotional faces, or investigating other elements of (social) reward processing such as reward anticipation.

Yet, the current study consisted of a unique sample of highly trauma-exposed male and female police officers with and without PTSD. This means that firstly, the wide range and high levels of trauma exposure, also in the control group, allowed us to investigate associations between alexithymia and trauma-exposure independently from – and in combination with – PTSD symptoms. As we did not include a non-trauma-exposed control group however, our conclusions are limited to trauma-exposed populations. Moreover, we only included police officers with or without PTSD that had no other comorbidities (except mild/moderate depression). Excluding other comorbidities, such as alcohol use disorder, that have also been associated with alexithymia (e.g. Cruise & Becerra, 2018), might limit the generalizability of our findings to PTSD patients with other comorbidities. On the other hand, limiting the comorbidities present in our sample might have reduced potential influence of confounding variables (e.g. long-term alcohol use) on our outcomes of interest, such as functional brain activity and brain structure. Secondly, the current sample advances our knowledge of alexithymia in first-responders and high-risk populations such as the police force. Although our findings might not generalise to other civilian trauma- and PTSD populations, first responders, especially females, are still largely understudied and an important group to take into account.

### 4.2. Clinical implications

The current findings emphasise the relevance of alexithymia in PTSD patients (Frewen et al., 2008a; Putica et al., 2021). Considering the potential negative impact of alexithymia on psychopathology course, treatment outcomes, and interpersonal relationships, it may prove fruitful to take alexithymia into account in psychotherapy (Eichhorn et al., 2014; Lumley & Norman, 1996; Morr et al., 2021; Ogrodniczuk et al., 2011; Putica et al., 2021; Taylor & Bagby, 2013; Zorzella et al., 2020). For instance, through mindfulness-based interventions, as a recent meta-analysis showed this might be effective in reducing levels of alexithymia (Norman et al., 2019). Also, additional (online) interventions can create an opportunity to practice dealing with emotions, and psychotherapy sessions supplemented with synthetic oxytocin administration have been shown to increase emotion detection and sharing, especially in individuals with high alexithymia (Quirin et al., 2014; Samur et al., 2013; Luminet et al). Another fascinating example showed amygdala neurofeedback could reduce levels of alexithymia in military trainees, in parallel to improved stress coping and reduced amygdala reactivity (Keynan et al., 2019). Such novel interventions may prove promising in prevention or treatment of alexithymia and related mental health problems in high-risk populations. Importantly, it is essential to consider that the mixed findings from both this, and previous studies (which investigated different samples, e.g. healthy participants, community-based PTSD patients or community-based trauma-exposed participants) emphasise the need for personalised risk prediction and treatment approaches.

## 5. Conclusion

To conclude, this study investigated the neurobiological correlates of alexithymia, in turn contributing to the understanding of alexithymia. We replicated the strong association between alexithymia and PTSD symptoms found in previous literature, although we did not substantiate associations between trauma-exposure and alexithymia, suggesting that alexithymia might not be directly associated to trauma, rather it may be indirectly associated, possibly as a predisposing risk factor for PTSD, or a coping mechanism specifically related to the increased psychopathological distress associated with PTSD. No significant associations were identified between alexithymia and short-term basal salivary oxytocin levels and functional reactivity of the amygdala to emotional faces, and insula reactivity to social reward. This adds new knowledge to the field, suggesting that the neurobiological mechanisms underlying alexithymia may differ from those investigated in this study. Although alexithymia was weakly associated with larger insula volumes, this did not survive correction for multiple comparisons and must be interpreted with caution. Future research into the neurobiological correlates of alexithymia might focus on oxytocin reactivity to social paradigms, tasks probing the recognition of participants’ own emotions, or investigation of functional connectivity networks that have been implicated with emotion recognition and alexithymia. Although we did not find any convincing neurobiological correlates of alexithymia in the markers investigated in this study, our findings advance the understanding of alexithymia in relation to trauma-exposure and PTSD symptoms, and once again emphasise the clinical relevance of alexithymia in PTSD.

## Supplementary Material

231020_Supplementary Material_v4_notannotated.docx

231020_Supplementary Material_v4_anonimized.docx

## Data Availability

The data that support the findings of this study, and summary statistics of the dataset are available on request from the corresponding author, Laura Nawijn. The data are not publicly available due to their containment of information that could compromise the privacy of research participants.
