# Supplementary material for: How does it feel? An exploration of neurobiological and clinical correlates of alexithymia in trauma-exposed police-officers with and without PTSD
Source: Eur J Psychotraumatol. 2023 Nov 21;14(2):2281187. doi: 10.1080/20008066.2023.2281187 (PMC10990451; doi:10.1080/20008066.2023.2281187)
Supplement: 231020_Supplementary Material_v4_anonimized.docx [file ZEPT_A_2281187_SM2339.docx]

# **Supplementary Material**

How does it feel? An exploration of neurobiological and clinical correlates of alexithymia in trauma-exposed police-officers with and without PTSD

*
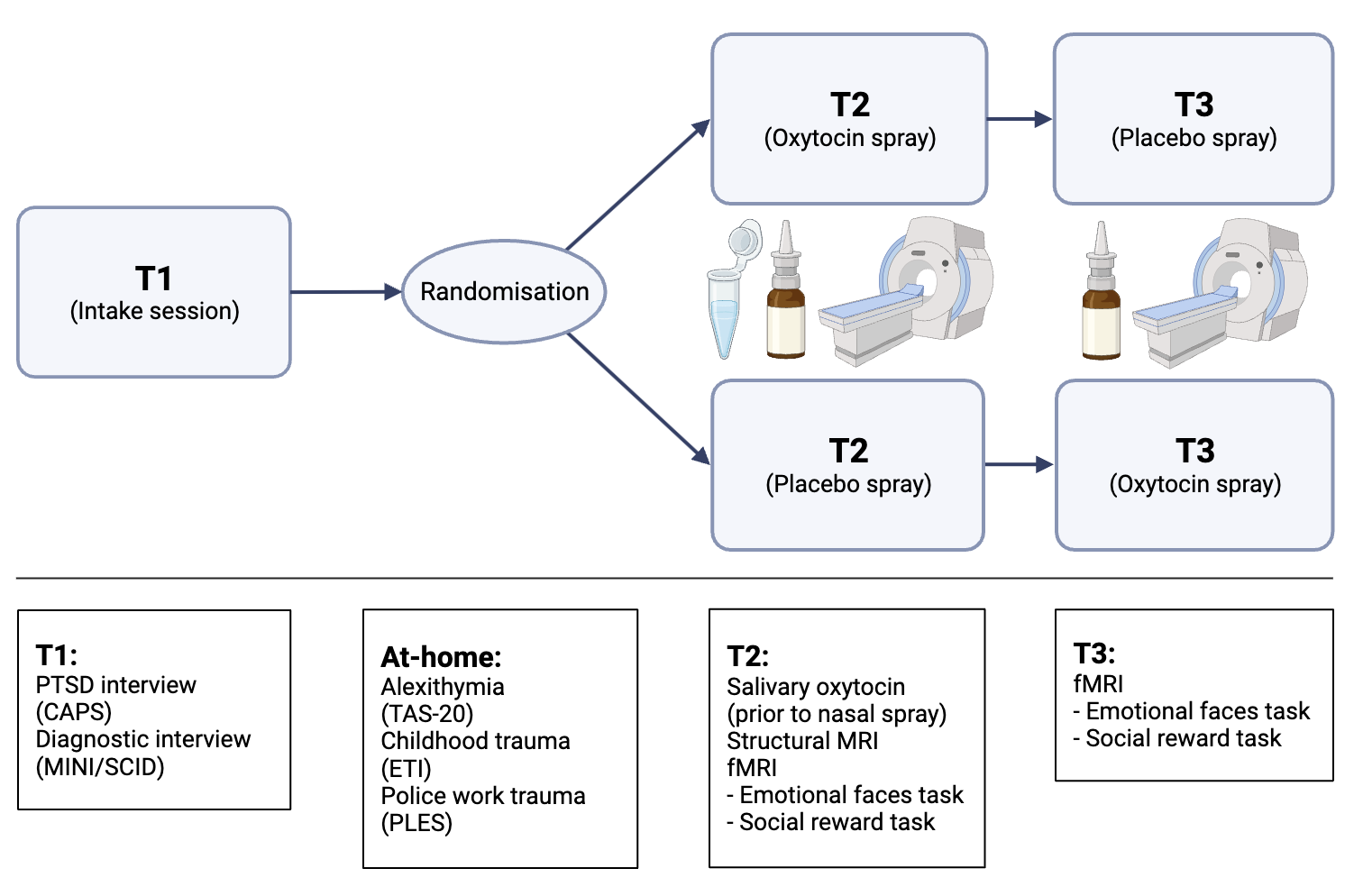
*

## **Supplementary Figure S1**.

Overview of the study protocol. During the intake session (T1), clinical interviews were taken including the Clinician Assessed PTSD Scale (CAPS) for PTSD symptoms and diagnosis, and the MINI or SCID diagnostic interviews for additional psychiatric diagnoses. After inclusion, participants filled in self-report questionnaires at home, including the Toronto Alexithymia Scale (TAS-20) for Alexithymia, the Early Trauma Inventory (ETI-SF) for childhood trauma, and Police Life Events Scale (PLES) for police-work-related trauma exposure. Participants were randomised to receive either oxytocin nasal spray (40 IU Syntocinon) at T2 and placebo nasal spray (saline, NaCl 0.9%) at T3, or vice versa. At T2, first a saliva sample was taken to assess endogenous oxytocin levels. Next, nasal spray administration took place, and subsequently the neuroimaging protocol was started, including a structural MRI scan and fMRI tasks (emotional face matching task, social reward task). At T3, participants received the other nasal spray administration, and subsequently the neuroimaging protocol was started, including the same fMRI tasks as performed at T2 (emotional face matching task, social reward task).

*Abbreviations*: CAPS, Clinician Administered PTSD Scale (Blake et al., 1995); MINI, Mini International Neuropsychiatric Interview (Sheehan et al., 1997; van Vliet et al., 2000); SCID, Structured Clinical Interview for DSM-IV (First et al., 2012; van Groenestijn et al., 1999); TAS-20, Twenty Item Toronto Alexithymia Scale (Bagby, Taylor, & Parker, 1994); ETI-SF, Early Trauma Inventory – Short form (Bremner et al., 2007); PLES, Police Life Events Scale (Carlier & Gersons, 1992); MRI, Magnetic resonance imaging; fMRI, functional magnetic resonance imaging, IU, International Units.

##

##

**
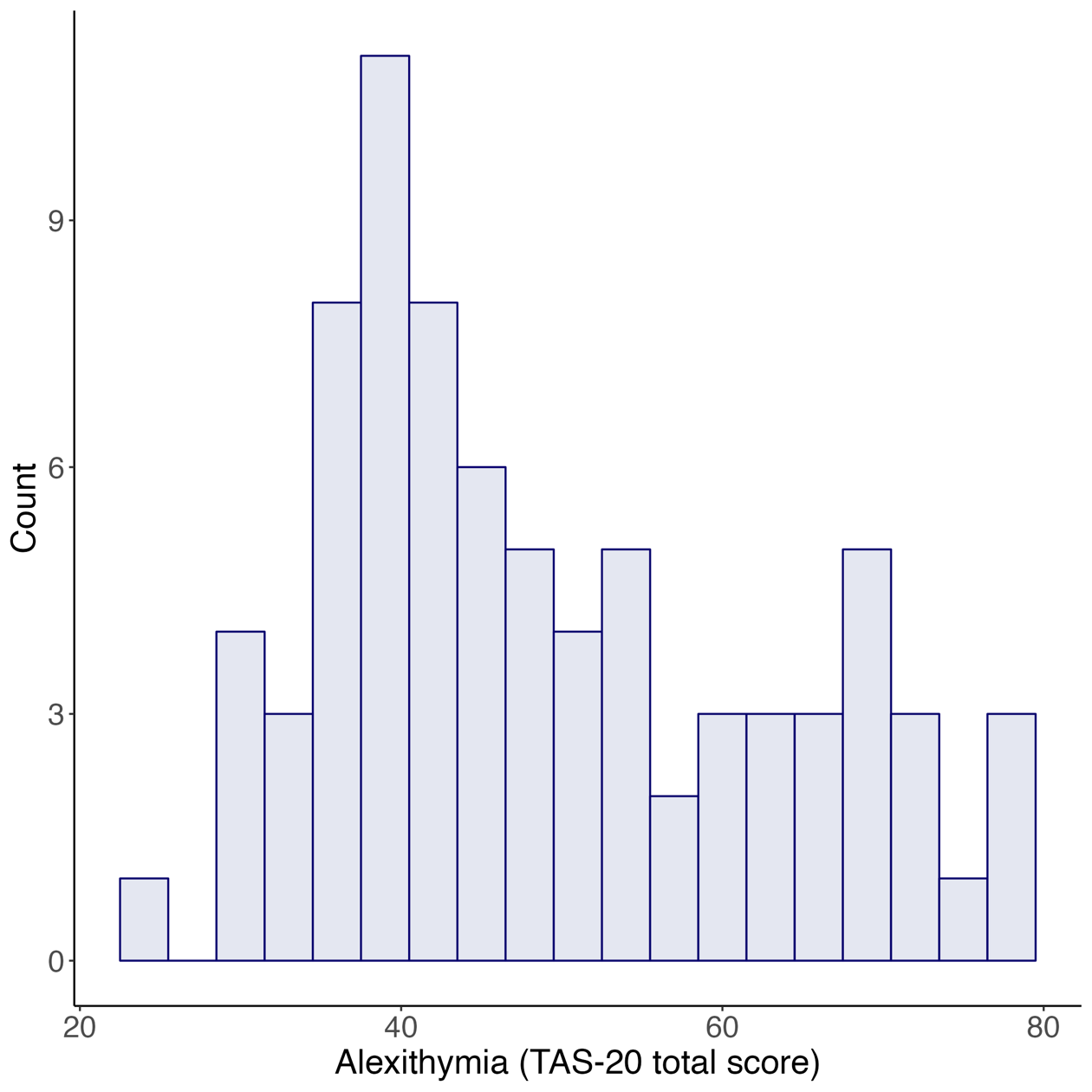
**

##

## **Supplementary Figure S2**.

Histogram of Alexithymia TAS-20 total scores.

#


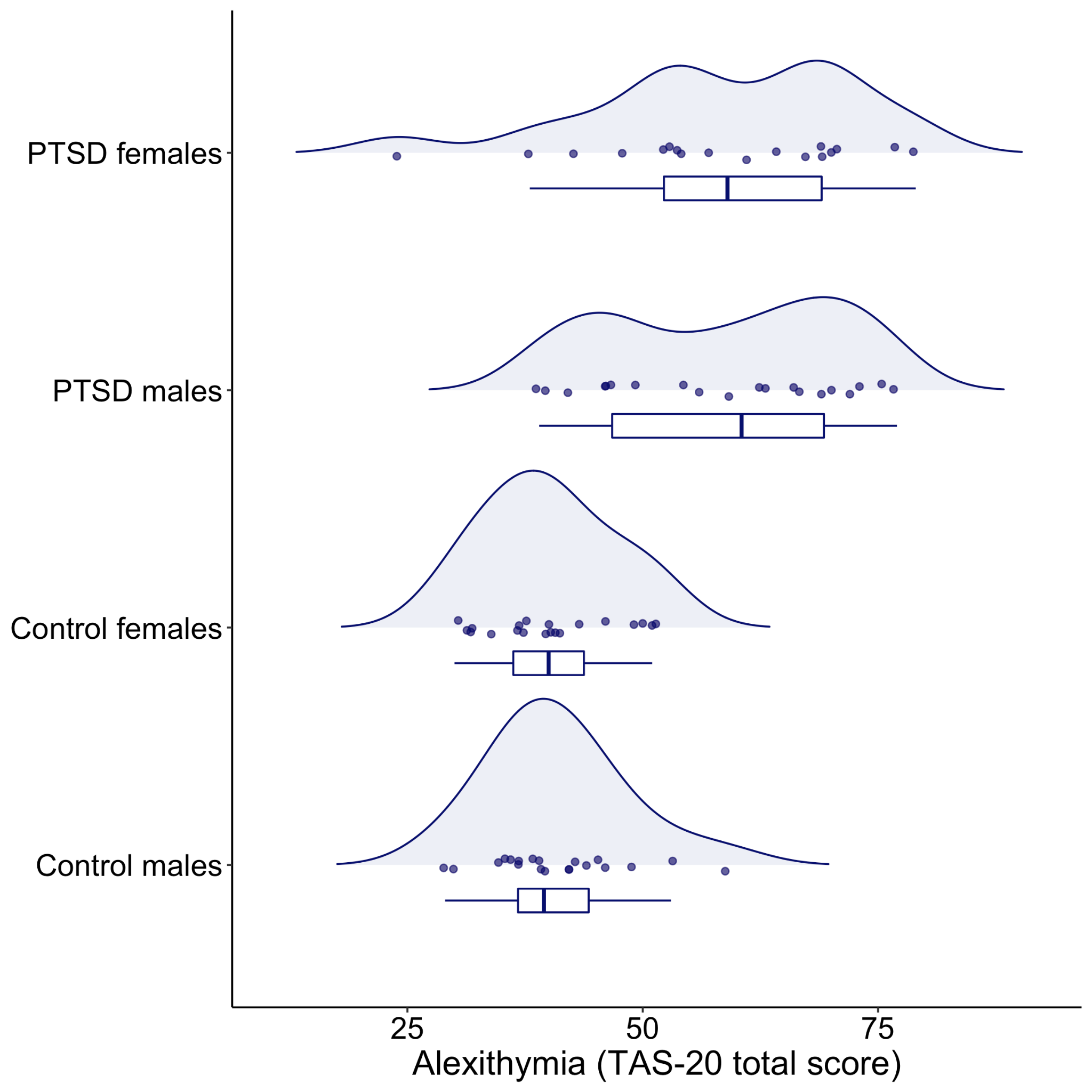


##

## **Supplementary Figure S3.**

Density- and boxplots of alexithymia (TAS-20) scores per PTSD diagnosis and sex group (black dots denote individual scores)

## **Supplementary Table S1.** Spearman's Rho correlation coefficients (⍴) between alexithymia and PTSD symptom subscales in PTSD patients (n=38)

| **PTSD symptoms (CAPS)** | |  | **Alexithymia (TAS-20)** |
| --- | --- | --- | --- |
|  | Total score |  | **0.497^**^** |
|  | Intrusions |  | 0.100 |
|  | Avoidance |  | 0.147 |
|  | Emotional numbing |  | **0.536**** |
|  | Arousal |  | **0.471**** |

*Note: ** Correlation is significant at the 0.01 level (2-tailed), * Correlation is significant at the 0.05 level (2-tailed).*

**
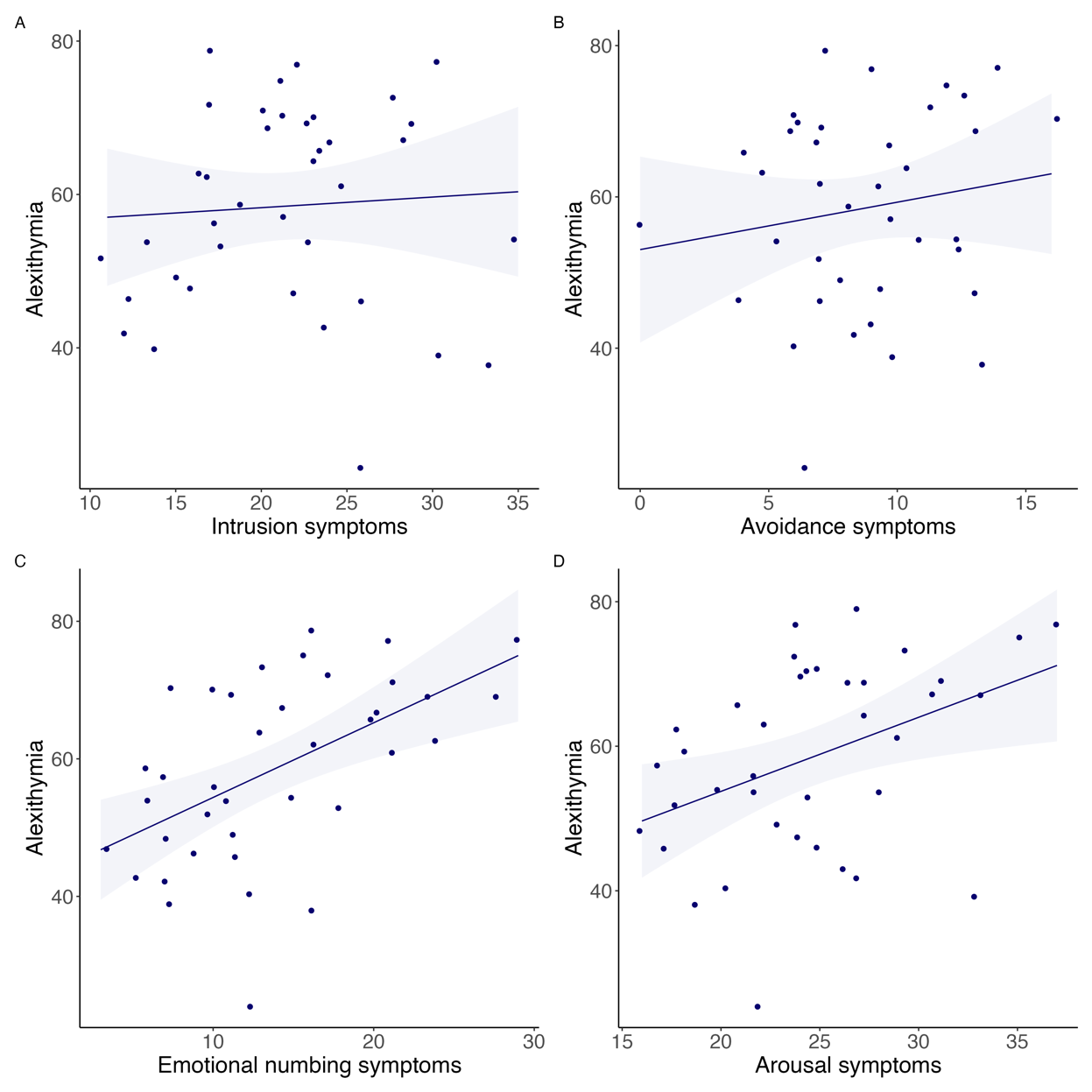
**

**Supplementary Figure S4.**

Scatter plots of linear associations between alexithymia & PTSD symptom severity subscales, within PTSD participants only (n=38). Panels display the associations between Alexithymia (TAS-20 total score) and A. PTSD Intrusion symptoms (total score on CAPS intrusion items; r(36) = 0.062, p = 0.710), B. Avoidance symptoms (total score on CAPS intrusion items; r(36) = 0.158, p = 0.344), C. Emotional numbing symptoms (total score on CAPS Emotional numbing items; r(36) = 0.534, p<0.001**), and D. Arousal symptoms (total score on CAPS arousal items; r(36) = 0.405, p = 0.012*). Plots include a linear regression line and 95% confidence intervals (light blue bands). ** Correlation is significant at the 0.01 level (2-tailed), * Correlation is significant at the 0.05 level (2-tailed).

**Supplementary Table S2.** Results of hierarchical regression analyses for the association between Alexithymia and Childhood trauma exposure (1A, n=78) and Police work-related trauma exposure (1B, n=77)

| **2A.** | **Childhood trauma (ETI-SF)** | | |
| --- | --- | --- | --- |
|  | β | | p |
| *Step 1.* |  | |  |
| Alexithymia (TAS-20) | 0.076 | | 0.509 |
|  |  | |  |
| *Step 2.* |  | |  |
| Alexithymia (TAS-20) | -0.073 | | 0.631 |
| PTSD diagnosis | 0.220 | | 0.150 |
|  |  | |  |
| *Excluded variables* |  | |  |
| Sex | -0.089 | | 0.439 |
| Age | 0.020 | | 0.865 |
| Education | -0.015 | | 0.896 |
| **2B.** | **Police-work related trauma (PLES)** | | |
|  | β | p | |
| *Step 1.* |  |  | |
| Alexithymia (TAS-20) | -0.107 | 0.355 | |
|  |  |  | |
| *Step 2.* |  |  | |
| Alexithymia (TAS-20) | -0.068 | 0.490 | |
| Sex | -0.350 | <0.001** | |
| Age | -0.436 | 0.049* | |
| Years of service | 0.793 | <0.001** | |
|  |  |  | |
| *Step 3.* |  |  | |
| Alexithymia (TAS-20) | -0.117 | 0.368 | |
| Sex | -0.353 | <0.001** | |
| Age | -0.470 | 0.041* | |
| Years of service | 0.832 | <0.001** | |
| PTSD diagnosis | 0.079 | 0.560 | |
|  |  |  | |
| *Excluded variables* |  |  | |
| Education | 0.039 | 0.697 | |

*β = standardized beta coefficients, TAS-20 = Toronto Alexithymia Scale*

*ETI-SF = Early Trauma Inventory Short form, PLES = Police Life Events Checklist*

*** Correlation is significant at the 0.01 level (2-tailed), * Correlation is significant at the 0.05 level (2-tailed).*

**Supplementary Table S3A.** Correlation coefficients (Spearman's ρ) between alexithymia, childhood trauma exposure (ETI-SF) and police work-related trauma exposure (PLES) in the entire sample (n=78)

|  |  |  | **Alexithymia** | | **Childhood trauma** | |  |  |  |  | **Policework trauma** | |
| --- | --- | --- | --- | --- | --- | --- | --- | --- | --- | --- | --- | --- |
|  |  |  | Total |  | Total | General | Physical | Emotional | Sexual |  | Total |  |
| **Alexithymia (TAS-20)** | |  |  |  |  |  |  |  |  |  |  |  |
|  | Total score |  | 1 |  | 0.080 | 0.022 | 0.048 | 0.218 | -0.042 |  | -0.099 |  |
| **Childhood trauma (ETI-SF)** | |  |  |  |  |  |  |  |  |  |  |  |
|  | Total score |  | 0.080 |  | 1 | 0.823** | 0.801** | 0.599** | 0.300** |  | 0.415** |  |
|  | General trauma |  | 0.022 |  | 0.823** | 1 | 0.460** | 0.316** | 0.103 |  | 0.378** |  |
|  | Physical trauma |  | 0.048 |  | 0.801** | 0.460** | 1 | 0.438** | 0.183 |  | 0.359** |  |
|  | Emotional trauma |  | 0.218 |  | 0.599** | 0.316** | 0.438** | 1 | 0.193 |  | 0.047 |  |
|  | Sexual trauma |  | -0.042 |  | 0.300** | 0.103 | 0.183 | 0.193 | 1 |  | 0.005 |  |
| **Police work trauma (PLES)** | |  |  |  |  |  |  |  |  |  |  |  |
|  | Total score |  | -0.099 |  | 0.415** | 0.378** | 0.359** | 0.047 | 0.005 |  | 1 |  |

*Note: ** Correlation is significant at the 0.01 level (2-tailed), * Correlation is significant at the 0.05 level (2-tailed).*

*TAS-20 = Toronto Alexithymia Scale; ETI-SF = Early Trauma Inventory – Short Form; PLES = Police Life Events Scale.*

## **Supplementary Table S3B.** Partial correlation coefficients between alexithymia, childhood trauma exposure (ETI-SF) and police work-related trauma exposure (PLES) in the entire sample (n=78), corrected for sex, age and PTSD diagnosis

|  |  |  | **Alexithymia** | | **Childhood trauma** | |  |  |  |  | **Policework trauma** | |
| --- | --- | --- | --- | --- | --- | --- | --- | --- | --- | --- | --- | --- |
|  |  |  | Total |  | Total | General | Physical | Emotional | Sexual |  | Total |  |
| **Alexithymia (TAS-20)** | |  |  |  |  |  |  |  |  |  |  |  |
|  | Total score |  | 1 |  | -0.093 | -0.105 | -0.057 | -0.107 | 0.002 |  | -0.068 |  |
| **Childhood trauma (ETI-SF)** | |  |  |  |  |  |  |  |  |  |  |  |
|  | Total score |  | -0.093 |  | 1 | 0.786** | 0.803** | 0.752** | 0.690** |  | 0.358** |  |
|  | General trauma |  | -0.105 |  | 0.786** | 1 | 0.485** | 0.439** | 0.285* |  | 0.431** |  |
|  | Physical trauma |  | -0.057 |  | 0.803** | 0.485** | 1 | 0.472** | 0.509** |  | 0.322** |  |
|  | Emotional trauma |  | -0.107 |  | 0.752** | 0.439** | 0.472** | 1 | 0.453** |  | 0.045 |  |
|  | Sexual trauma |  | 0.002 |  | 0.690** | 0.285* | 0.509** | 0.453** | 1 |  | 0.198 |  |
| **Police work trauma (PLES)** | |  |  |  |  |  |  |  |  |  |  |  |
|  | Total score |  | -0.068 |  | 0.358** | 0.431** | 0.322** | 0.045 | 0.198 |  | 1 |  |

*Note: ** Correlation is significant at the 0.01 level (2-tailed), * Correlation is significant at the 0.05 level (2-tailed).*

*TAS-20 = Toronto Alexithymia Scale; ETI-SF = Early Trauma Inventory – Short Form; PLES = Police Life Events Scale.*

**Supplementary Table S4.** Results of hierarchical regression analyses for the association between Alexithymia and Endogenous Salivary Oxytocin Levels (n=75)

|  | **Oxytocin levels** | |
| --- | --- | --- |
|  | β | p |
| *Step 1.* |  |  |
| Alexithymia (TAS-20) | -0.164 | 0.161 |
|  |  |  |
| *Step 2.* |  |  |
| Alexithymia (TAS-20) | -0.143 | 0.353 |
| PTSD diagnosis | -0.032 | 0.837 |
|  |  |  |
| *Excluded variables* |  |  |
| Sex | -0.019 | 0.869 |
| Education | -0.055 | 0.638 |
| Hormonal status | 0.043 | 0.710 |

*Note,* β *= Standardized beta coefficients, ** Correlation is significant at the 0.01 level (2-tailed), * Correlation is significant at the 0.05 level (2-tailed).*

**Supplementary Table S5.** Results of hierarchical regression analyses for the association between Alexithymia and Amygdala (n=71) and Insula Reactivity (n=71)

|  | **Amygdala reactivity** | |  | **Insula reactivity** | |
| --- | --- | --- | --- | --- | --- |
|  | β | p |  | β | p |
| *Step 1.* |  |  |  |  |  |
| Alexithymia (TAS-20) | -0.175 | 0.135 |  | -0.170 | 0.158 |
|  |  |  |  |  |  |
| *Step 2.* |  |  |  |  |  |
| Alexithymia (TAS-20) | -0.088 | 0.579 |  | -0.580 | 0.153 |
| PTSD diagnosis | -0.130 | 0.409 |  | -0.566 | 0.001** |
|  |  |  |  |  |  |
| *Excluded variables* |  |  |  |  |  |
| Sex | 0.147 | 0.208 |  | -0.077 | 0.521 |
| Education | -0.133 | 0.256 |  | 0.171 | 0.150 |

*Note,* β *= Standardized beta coefficients, ** Correlation is significant at the 0.01 level (2-tailed), * Correlation is significant at the 0.05 level (2-tailed).*

**Supplementary Table S6.** Results of hierarchical regression analyses for the association between Alexithymia and Bilateral Amygdala (n=74) and Insula Volume (n=74)

|  | **Amygdala volume** | |  | **Insula volume** | |
| --- | --- | --- | --- | --- | --- |
|  | β | p |  | β | p |
| *Step 1.* |  |  |  |  |  |
| Alexithymia (TAS-20) | 0.146 | 0.209 |  | 0.222 | 0.016* |
| Intracranial volume | 0.200 | 0.087 |  | 0.623 | <0.001** |
|  |  |  |  |  |  |
| *Step 2.* |  |  |  |  |  |
| Alexithymia (TAS-20) | 0.166 | 0.286 |  | 0.364 | 0.003** |
| Intracranial volume | 0.201 | 0.087 |  | 0.632 | <0.001** |
| PTSD diagnosis | -0.030 | 0.844 |  | -0.216 | 0.070 |
|  |  |  |  |  |  |
| *Excluded variables* |  |  |  |  |  |
| Sex | 0.089 | 0.618 |  | -0.228 | 0.097 |
| Education | 0.009 | 0.938 |  | 0.118 | 0.204 |

*Note,* β *= Standardized beta coefficients, ** Correlation is significant at the 0.01 level (2-tailed), * Correlation is significant at the 0.05 level (2-tailed).*
